# A Case of Ano-Rectal Abscess

**Published:** 1880-02

**Authors:** E. A. Cobleigh

**Affiliations:** Athens, Tenn.


### A CASE OF ANO-RECTAL ABSCESS.

By E. A. COBLEIGH, M.D., Athens, Tenn.

These affections are sufficiently common to fall under the observation of every practitioner, and, in timid or hesitating hands, not infrequently give rise to severe and extensive complications, often resulting in troublesome fistulæ. Among several which it has been my lot to manage, one case particularly interested me, notwithstanding that there was nothing very remarkable about it except perhaps its extent. But it confirmed the value of the precept of prompt interference in these cases, which I had learned and acted on previously, but which, for the once, I allowed myself to be tempted into neglecting. I give the details as noted at the time, and as I reported them soon after discharging the patient, to the *Hiwassee Medical Society*.

Just after dinner on November 5th, 1879, I was requested to call and see a child of L. D——. Found the patient to be a little girl, seven years of age, lying on her bed with a high fever, but complaining of no pain or other suffering. Had been ill three days, feverish for thirty-six hours, the pyrexia being reported worse about nightfall, but had kept her feet till to-day. Her mother at once exposed her person, and called my attention to a tender and swollen condition of the right side of the perineum, extending behind the anal opening, but giving no sense of higher heat than the rest of her feverish body, and not in the least red. Her bowels were acting regularly and without discomfort, stools normal except once or twice during the past three days, when a very slight mucoid admixture had been observed in the feces. Swelling had slowly but steadily increased from its incipiency, but was neither painful nor inconvenient, except to-day, on motion of the body or limbs; so the little patient lay quiet as a lamb in any position she was placed by the attendants. The girl seemed generally healthy and in fine bodily condition, aside from the threatening abscess—no scrofulous tendency, no hemorroids, and no evidence of any previous rectal disturbance. I could ascertain no cause for the present trouble, and thought it might not prove troublesome, especially as the tumefaction was so slight and no discoloration or heat of skin had occurred. There was not even an indistinct sense of fluctuation. As her bowels were acting freely already, I put her at once on opiates, without premising a saline cathartic. Locally, I ordered lead and laudanum fermentations, or cold lotions of the same, as might prove most grateful to the child. I also explained the nature of the affection to her parents, and warned them of a possible contingency which I had several times witnessed, anal fisule.

On the next day (6th) found her condition not materially changed, no seeming increase of tumefaction, fever slight, no fluctuation, but the affected spot evidently more sensitive to my touch. Passed a rather restless night and had very high fevers from dark to midnight, so her mother said. Still complains of not a particle of pain. Bowels still free and stools natural and painless. Knowing the treacherous nature of these abscesses, my suspicions of the case were by no means quiescent, and yet I hesitated to express them, or resort to the knife, mainly because neither redness, deep fluctuation nor pain presented themselves. Treatment continued.

Nov. 7th. Patient begs not to be moved to-day; lies in semi-prone position; passed a bad night despite the opiates; swelling some greater; skin now slightly reddened and hot; tumefaction very tense and closes the anus, obliterating sulcus between nates, extending to the right tuber ischii. Reported very feverish for six hours during last night. Was on the point of lancing the now fully formed abscesses, when my hand was stayed by the child's father, and I was begged to desist. I insisted on no further delay in opening it, explaining the hazard of dallying, and was proceeding again to use the knife, when the father once more held my hand and forbade it. I refused to treat the case in any other manner, stating my decision firmly but kindly, and left the house. My patient even now asserts that she suffers no pain or throbbing at all, only a feeling of tension and weight.

Nov. 8th. Was sent for just after dark last night, but was out of town. Got home late in night and was almost immediately summoned to the country again. On returning, I visited a couple of patients in town before going home—now about noon—and although three urgent calls had come for me to see little Mamie since daylight, I got neither message till I reached my house for dinner. Proceeded at once to her beside, and found that she had had a terrible night of it; high fever, rigors, intense pain, nearly throwing her into convulsions, and causing her to writhe and scream with agony. Poultices had been applied by the family, and opiates given with a free hand. About two hours before my present visit she had got easy, become quiet, and fallen asleep, still rigidly maintaining the semi-prone position occupied by her yesterday. She awoke with a start on my attempting gentle digital examination of the now mammoth swelling, extending down the thigh, over the nates and even up the sacral region. The inguinal glands were sympathetically enlarged and tender. Skin over tumefaction wore a deep glossy erysipelatous hue, and was exquisitely sensitive. Fluctuation marked. Having now become tranquil, the child's parents desired me to go away again, let her alone, and leave the "bealing" to break of itself; but before they could prevent it, I plunged my lancet through the tense skin to the depth of nearly an inch, and quickly slit up the parts to the extent of about an inch and a half, at the side of the anus and about three-fourths of an inch from the opening. This procedure resulted in the forcible gushing forth of a large quantity of horribly offensive pus, and a decided decrease in the size of the previous tumefaction. It continued to discharge freely for some minutes, and during the next twenty-four hours not less than a pint and a half of purulent matter escaped. On probing the wound with my finger a very capacious pouch was demonstrated to exist, extending along the whole lateral perineal region, around the rectum to beyond the median line, and denuding fully one-half of the gut so completely that it gave the impression of a smooth, fleshy cylinder, alout one and a half inches long, passing through the diseased part. I now ordered cold hop poultices applied to absorb the discharge, and the free use of a weak solution of potassium permanganate, by means of a syringe, to the interior of the abscess, and administered morphia hypodermically, as my patient was screaming violently from mingled pain and fright. Also placed her in a position to facilitate free drainage. Next morning found her perfectly easy, free from fever, and bowels acting from a dose of oil given about daylight. Discharge is measurably diminishing from hour to hour. At night I drew edges of incision firmly together by plaster, but left room for drainage, though only slight oozing was going on. I then adjusted compresses to obliterate cavity of abscess. In twenty-four hours all discharge had ceased, adhesion was going on to perfection, bowels in good order, the patient up and about, swelling disappeared, and case was discharged. No fistulæ resulted at any point, either opening outwards or into the gut.

There are no features of special interest connected with this case except such as are clearly apparent from a review of the history already detailed. 1st. The extensive tumefaction ; 2d. The lack of pronounced symptoms of high inflammatory action, eventuating in pus, until some time after a considerable amount of pus had doubtless already formed, giving the disease a latent character, and throwing me, for once, and only once, off my guard; 3d. The rapid increase of purulent matter towards the last, burrowing so widely in every direction; 4th, The regular, yet painless, stools, until the anus became mechanically closed; 5th. The dissection of the rectum out of its bed without any sympathetic rectitis, and with no ultimate connection being established between the pus depot and bowel. Had this been a scrofulous abscess, none of these things would have been surprising, but the acuteness and rapid course of my case disproved any such a supposition; nor did the patient give any strumous indications, while the character of the pus was by no means indicative of constitutional taint. I attempt no excuse for allowing myself to delay in freely opening this abscess at an early stage, for such has always been my habit in other instances; and had there been any pain or throbbing in the present case, no doubt that rule would have continued to guide me. However, my patient recovered nicely, notwithstanding my own tardiness of action and the extent of her disease, and for that I am of course grateful; the rapidity and completeness of her recovery astonishing myself but slightly less than it did her friends.

Though ano-rectal abscesses occur at all ages, this was the youngest patient I ever saw thus affected. They generally arise from the strumous diathesis, extension of neighboring inflammations, or direct injury. This case probably resulted from some trivial and unremembered bruise or fall. They are exceedingly liable, especially the strumous, to eventuate in fistulæ. When scrofulous in nature, prompt evacuation is the only reliable means of preventing them from pointing into the bowel and doing mischief, and early interference is best in all cases. They not seldom lie deeply, necessitating a bold lance and fearless division of the overlying tissues. Give them always a large vent. I have personally never succeeded in aborting a case, but this may be because no one ever fell into my hands for treatment until it had been under headway for two or three days. Once fully formed, the very nature of the extensive cellular structures pervading the perineum and neighboring regions favors, as in the foregoing case, very wide burrowing of pus and mischievous damage to these loose and delicate tissues, often ending in long, tortuous fistulæ with openings remote from the primal seat of inflammation. I hope the narration of this case may point out the dangers of delay to others as forcibly as it has reminded me thereof, for it is not every ano-rectal abscess that would, under like circumstances as those I have detailed, have resulted in so complete, speedy, and satisfactory a recovery

